# Death from Anæsthesia

**Published:** 1861-05

**Authors:** 


### Selections.

DEATH FROM ANÆSTHESIA.—POST-MORTEM INDICATIONS. —*Lecture VI.*, *Tuesday*, *June* 5, 1860.—After a few allusions to the subjects of his last lecture, the Lecturer passed to describe those symptoms which denote a fatal result during the administration of chloroform. These may be divided into two distinct heads, viz., syncope and coma.

Death from coma very seldom takes place , not more than about twelve persons have died from this class of death. The symptoms of coma are apoplectic in their nature. There is very little reason to doubt but that the whole of those persons who have died in the comatose state had preëxistent disease of the kidneys. In disease of either liver or kidneys, we should be especially careful in the administration of chloroform.

In death by syncope under chloroform, the event takes place suddenly, sometimes within the first eight or ten inspirations: in one case, that occurred at St. George's Hospital, the patient is said to have died on taking the first sniff of the chloroform. Dr. Richardson is of opinion that in the last mentioned case the patient did not die from the effects of the chloroform, but from fright. He noticed cases of patients dying on the operating table from fright before they were touched with the knife, long before chloroform was known.

Death in this acute mode generally occurs at about the end of the first degree; and a very curious circumstance connected with it is, that up to the last moment there is no indication of a fatal result. The patient seems to be progressing favorably ; there is no difficulty in the breathing, and the very best and purest chloroform may be in use ; suddenly there is a call that the pulse has stopped—it has not gradually slacked or fluttered, but it has stopped at once and abruptly under the finger—and if it does not commence pulsating within from five to ten seconds, there is little chance of saving the patient's life. The cause of this stoppage of the pulse is, of course, owing to paralysis of the heart ; but here, again, is a circumstance to be noted—it is the right (or venous) side of the heart that becomes paralysed primarily, and not the left

(or arterial). The right side being paralysed, the blood is prevented from passing to the left side of the heart; the left ventricle contracts and expels the blood contained in it through the arterial system, but finding no further blood to contract upon, the arteries come to a full stop, and death is the result; for, according to the law of hydrostatics, a double column of blood (one arterial and one venous) is required in constant circulation for the maintenance of life. After the pulse has stopped, the patient often gives a shudder and is convulsed (the same convulsive action occurs in animals when bled to death), and sometimes the respiratory organs make a few efforts to continue their functions; but so sudden is life stopped, that it is as if the great aorta were tied, and the blood prevented from making its circulation. The lips turn of a pallid color before death takes place.

In the case of a patient showing comatose symptoms, immediately remove the chloroform, and give plenty of fresh air. A stimulant may be administered occasionally, and some recommend a mild emetic if the patient seems inclined te vomit; but the best plan is to leave Nature to herself. Some recommend the holding of ammonia under the nose, but Dr. Richardson does not: for though it might produce no harm to hold it to the nose for one minute, yet it would do no good whatever, and we are to remember that ammonia, when inhaled in excess, is an anæsthetic itself.

If there are signs of a patient's dying by syncope, withdraw the chloroform in the first place, give plenty of fresh air, and throw the clothes loose: do not administer ammonia nor throw water upon the chest; but if the breathing stops, try to start it by means of artificial respiration. Dr. Richardson here explained the different methods in use for producing the desired end. The readiest plan is that introduced by Dr. Marshall Hall, and has received the name of the "Ready Method." It is effected by laying the patient on his side, and rolling him over on his abdomen, then back again, keeping up a steady alternate motion. But this plan, although answering very well for children, does not at all well for adults, owing to the strength of the abdominal muscles and the stiffness of the ribs. An adult requires to inhale no less than twenty cubic inches of air at each inspiration to enable him to live; but by this means not more than ten cubic inches can be taken in at once by a dead adult subject.

Mr. Reid, of Regent street, invented an apparatus for artificial respiration. It consisted of two tubes, which were placed one in each nostril, the other ends of these tubes fitting into a piston, with valves so arranged that one tube threw air into the lungs, and the other drew air out, alternately. The inventor used to close his mouth, and be fed with air through the nose, by way of illustrating the effects of his apparatus.

Dr. Marcet has also invented a very ingenious instrument, but it is too complicated for general professional use; it might do very well for an hospital. Its principle was somewhat similar to the instrument of Mr. Reid.

Dr. Richardson has also invented a machine for this purpose, very simple, effective, and powerful. It has the great advantage of portability, as it can be conveniently carried in the coat-pocket. It consists of a double-acting pair of bellows, arranged with valves, so that by working one handle we draw air from the lungs, and by working the other we drive fresh air into its place. By these bellows no less a quantity than thirty cubic inches of air can be administered to or extracted from the lungs by one movement of the handle. (This instrument was passed round for inspection.)

There is a curious incident connected with artificial respiration: it is, that if when an animal is dying, we use the bellows a little, so as to keep up the respiration, we keep up life for some little time, but the moment we leave off its use, the animal dies. In using the bellows, it is well never to give too much air at each successive stroke, as cases have been known of the air-cells being ruptured through excess of air, administered in this manner. It is of little use keeping up artificial respiration any longer than fifteen minutes.

A plan has been suggested for setting the heart in motion by galvanism. If we apply the galvanic current to a portion of muscle within from one to three hours after death, the muscle will often contract; and if we use an electro-magnetic machine, we can produce a series of contractions. But Aldini, Volta, and all the great galvanists, admit that the heart does not act like other muscles; indeed, the true rhythmical action of the heart can not he reproduced by galvanism except directly after death.

There have been many violent discussions as to the action of galvanism, and it still remains a great question whether it communicates a new force entirely, or whether it merely calls up the remains of power that previously existed in the muscle. This point has never yet been decided; but supposing that by the aid of galvanism we could set the heart in true rhythmical action, the great question yet remains to be solved, how is the current to be applied? It has been tried in every way that could be thought of: it has been applied through the medium of the nerves, it has been applied to the pneumogastric nerve, and Dr. Snow on one occasion pushed two needles through the wall of the chest right into the muscular wall of the heart; but in no one of the numerous experiments that have been made has action of the heart been produced. Dr. Richardson has tried numbers of experiments with a like result. In one experiment he killed a dog, and opened the jugular vein, and passed one galvanic wire through it into the heart; on applying the other pole to the chest, he could perceive no action of the organ; he then opened the chest, and applied the wire to the wall of the heart, with no better success; and he is of opinion that galvanism does more harm than good, since he has found by experiment that if he exposed two hearts of different animals, and applied galvanism to the one and let the other alone, the one that he had galvanised always stopped beating before the other one that was not galvanised. We may apply galvanism, but he (Dr. R.), speaking with a fair knowledge of its properties, could think of no applicable method of making it useful in exciting contraction of the heart after death.

The post-mortem appearances in the bodies of persons and animals who have died from the administration of anæsthetics were next considered.

After death from chloroform, the lungs, heart, abdominal viscera, and kidneys are sometimes found to be greatly congested, both in inferior animals and in the human subject. Sometimes the brain and spinal cord are also congested; at others the lungs are of a milk-like whiteness, the veins and the right side of the heart full of blood, and the arteries and left side empty. Dr. Richardson has seen the post-mortem effects of narcotics in as many as 2,000 inferior animals; and in some cases, after chloroform, he has occasionally found that when death took place suddenly, there were spots of congestion in the lungs, but no change whatever in them sufficient to account for death. Usually the lungs were bloodless, and there were little evidences of change in the nervous system; the great and only cause of death being paralysis of the heart.

Amylene leaves the same effects as chloroform on the human subject. Ether produces more decided congestion of the lungs and brain.

The Lecturer then proceeded to destroy a rabbit by means of a strong dose of chloroform, and opened the animal to show his auditors practically the post-mortem appearances. The experiment was highly successful, and the theory that death is produced by paralysis of the heart was shown; for while the lungs were free of any congestion, the right side of the heart was gorged with blood, and the left side empty.

Finally, Dr Richardson expressed his regret that the course of Lectures on Anæsthesia and Anæsthetics had come to an end, and thanked the members for the deep interest and attention they had shown to the subject of the lectures. As this was the first course of lectures that he had delivered upon anæsthesia, and indeed the first course that had been delivered anywhere, he had found some difficulty in arranging his progress from step to step; he had been undecided how to make the beginning, but the plan he had adopted had proved sufficiently practicable for his purpose. He hoped that some of his auditors might be induced to make the subject of anæsthesia a part of their studies. In conclusion, he expressed his best thanks to Mr. Lloyd Bullock for his kindness in doing everything in his power to procure specimens for these lectures, and to Mr. Vacher (Mr. Bullock's assistant) for his very able assistance.

Mr. President Waite then rose, and in a brief but able speech requested the members and gentlemen present to join him in a vote of thanks to Dr. Richardson. The vote was carried unanimously, amidst loud and prolonged acclamations. —*Dr. Richardson's Lectures, reported for the Dental Review.*

